# Extrinsic arterial compression and lower extremity ischemia after iliac vein stent placement: case report, review of literature

**DOI:** 10.1186/s42155-023-00358-x

**Published:** 2023-03-07

**Authors:** Mohammad Elsayed, Debkumar Sarkar, Ken Zhao, Yolanda Bryce, Adie Friedman

**Affiliations:** Interventional Radiology Service, Department of Radiology, Memorial Sloan Kettering Cancer Center, 1275 York Ave, New York, NY 10065 USA

**Keywords:** Iliocaval stenting, Iliac vein stenting, Extrinsic arterial compression, Arterial ischemia, Venous stent, Pelvic malignancy

## Abstract

**Background:**

Lower extremity ischemia due to extrinsic arterial compression by venous stent placement is a rare but increasingly recognized occurrence. Given the rise of complex venous interventions, awareness of this entity is becoming increasingly important to avoid serious complications.

**Case Presentation:**

A 26-year-old with progressively enlarging pelvic sarcoma despite chemoradiation developed recurrent symptomatic right lower extremity deep venous thrombosis due to worsening mass effect on a previously placed right common iliac vein stent. This was treated with thrombectomy and stent revision, with extension of the right common iliac vein stent to the external iliac vein. During the immediate post-procedure period the patient developed symptoms of acute right lower extremity arterial ischemia including diminished pulses, pain, and motorsensory loss. Imaging confirmed extrinsic compression of the external iliac artery by the newly placed adjacent venous stent. The patient underwent stenting of the compressed artery with complete resolution of ischemic symptoms.

**Conclusions:**

Awareness and early recognition of arterial ischemia following venous stent placement is important to prevent serious complication. Potential risk factors include patients with active pelvis malignancy, prior radiation, or scarring from surgery or other inflammatory processes. In cases of threatened limb, prompt treatment with arterial stenting is recommended. Further study is warranted to optimize detection and management of this complication.

## Background

Lower extremity ischemia due to extrinsic arterial compression by adjacent venous stents is a rare occurrence. Given the rise of complex venous interventions, awareness of this entity is becoming increasingly important to avoid serious complications including amputation. This case report describes a patient with pelvic sarcoma who developed lower extremity ischemia after the external iliac artery became compressed by an adjacent venous stent. Existing literature including risk factors and potential mechanisms are highlighted.

## Case presentation

A 26-year-old with progressively enlarging spindle cell sarcoma of the right pelvis despite multiple rounds of systemic and radiation therapy developed deep venous thrombosis and pulmonary embolism due to mass effect on the right iliac vein. This was treated with mechanical thrombectomy, inferior vena cava (IVC) and bilateral kissing common iliac vein stents, as well as initiation of therapeutic anticoagulation. The patient responded well until approximately two months after the procedure, where he presented to the emergency department with 1 week of worsening lower extremity swelling and pain. Physical exam was notable for diffuse pitting edema of the right lower extremity. Although the swelling limited mobility, there were no focal motor or sensory deficits. The extremity was warm and well perfused with 2 + doppler signals from the popliteal, posterior tibialis, and dorsalis pedis arteries. The left lower extremity was normal.

**Fig. 1:**
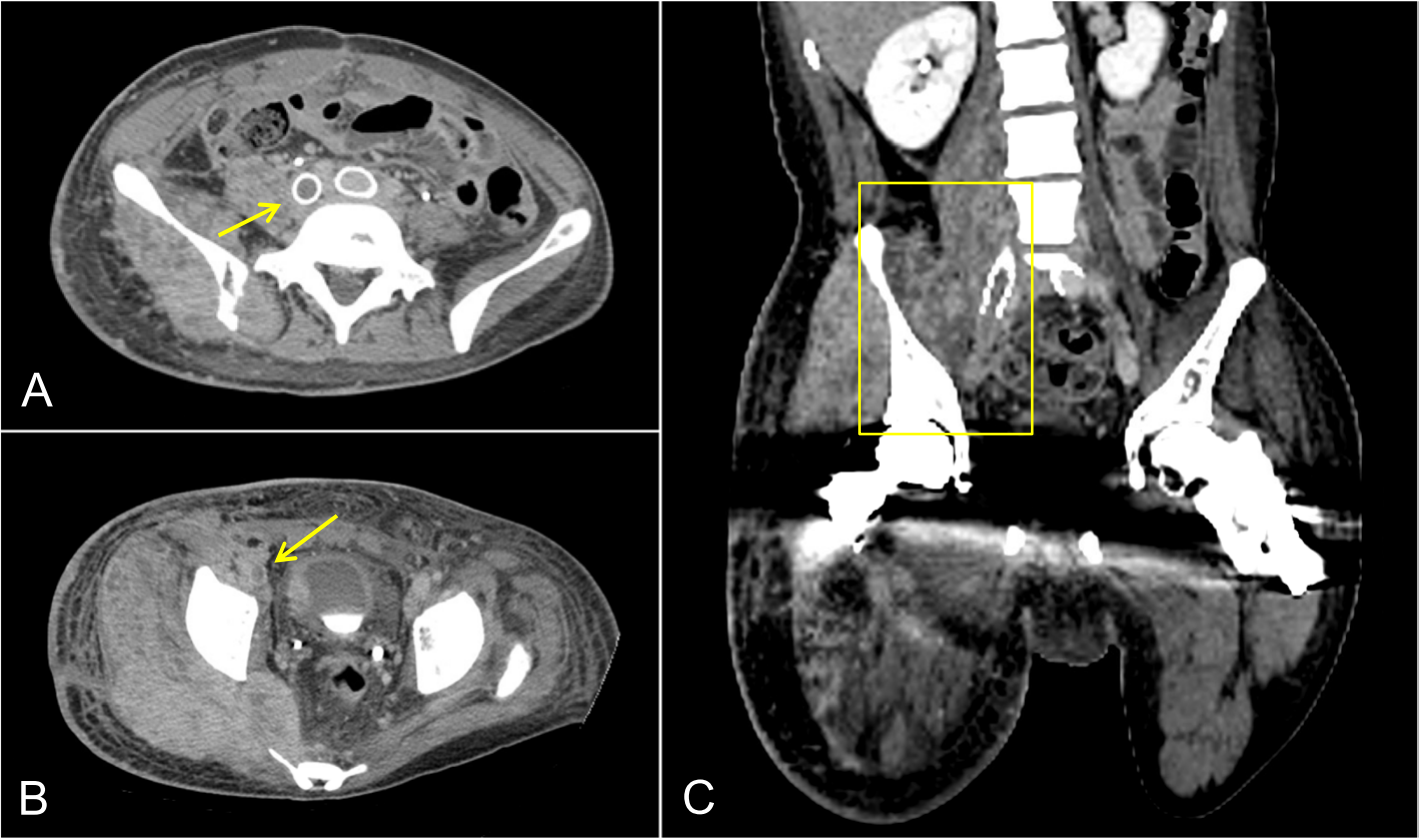
**A** and **B** Axial CT venogram demonstrates recurrent thrombosis of the right common iliac vein stent with extension of thrombus to the right lower extremity (arrows). **C** Coronal reformat of the CT venogram depicts tumor extension and mass effect on the right pelvic veins (box)

Right lower extremity ultrasound demonstrated acute deep venous thrombosis of the common femoral vein extending to the calf veins. Computed tomography (CT) venogram demonstrated thrombus throughout the right iliac vein and IVC stents with compression of the external iliac vein by the enlarging pelvic mass (Fig. 1). Given the patient’s severe symptoms, a decision was made to perform pharmacomechanical thrombectomy and stent revision.

**Fig. 2:**
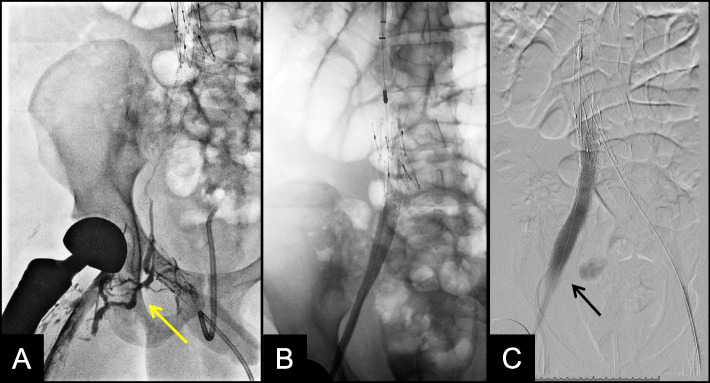
**A** Right pelvic venogram prior to iliocaval stent revision demonstrates extensive thrombus with minimal flow to the IVC and filling of pelvic venous collaterals (arrow). **B** Venogram after thrombectomy and stent revision demonstrates minimal residual thrombus with return of brisk flow through the IVC. **C** Digital subtraction venogram highlights the newly placed 14 mm Viabahn VBX stent along the external iliac vein stent (arrow)

While in the interventional radiology suite, posterior tibial vein access was obtained to infuse alteplase and perform venography, which demonstrated flow-limiting thrombus extending from the right popliteal to the common iliac vein. There was minimal flow to the IVC with extensive filling of collaterals (Fig. 2). The popliteal and right internal jugular veins were accessed and pharmacomechanical thrombectomy was performed using the Inari ClotTriever and FlowTriever Devices (Inari Medical, Irvine, CA, USA). After thrombectomy, venography demonstrated improved flow through the existing stents, but with flow-limiting stenosis of the external iliac vein. The existing right common iliac vein stent was extended to the external iliac vein using a Gore Viabahn VBX covered stent which was balloon dilated up to 14 mm. Final venography demonstrated brisk flow through the bilateral iliac veins and IVC (Fig. 2). Therapeutic lovenox was initiated.

While in the recovery unit, the patient’s leg appeared mottled. Bedside doppler revealed diminished signals in the right popliteal, posterior tibialis, and dorsalis pedis arteries. After approximately 4 h the patient reported pain, decreased sensation to the dorsum of the foot, and impaired motor movement of the toes and ankle.

**Fig. 3:**
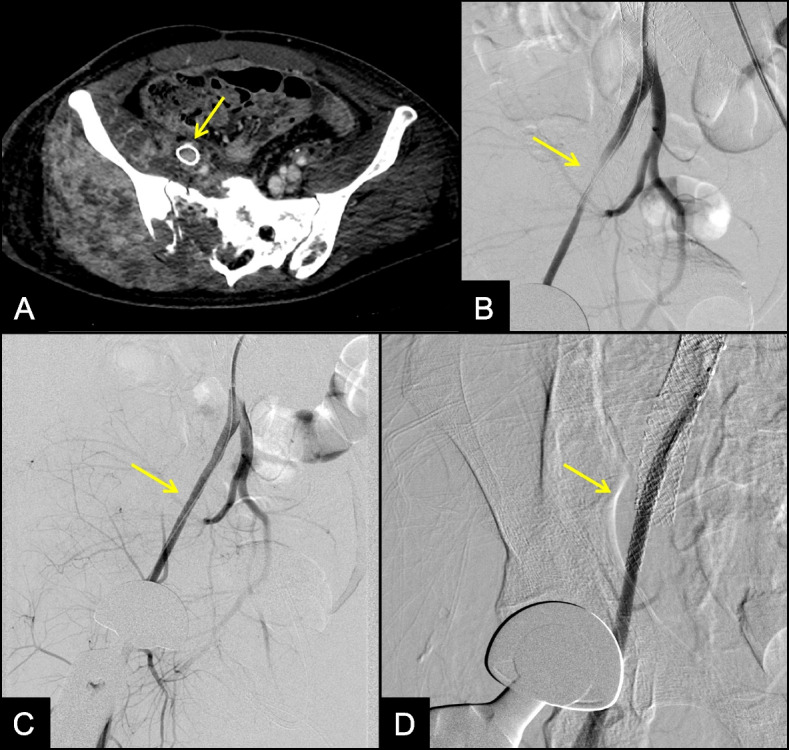
**A** Axial CT angiogram demonstrates high gradestenosis of the external iliac artery adjacent to the newly placed external iliac vein stent (arrow). **B** Angiogram through a 7 French sheath within the right common iliac artery demonstrates high grade narrowing of the external iliac artery secondary to compression from the adjacent venous stent (arrow). **C** and **D** Angiogram after placement of two overlapping 5 mm Viabahn VBX stents demonstrates resolution of the stenosis with brisk flow (arrows)

A CT angiogram was performed, demonstrating high grade narrowing right external iliac artery adjacent to the newly placed right external iliac vein stent (Fig. 3). There was distal reconstitution with otherwise patent lower extremity vessels. Non-invasive vascular studies demonstrated a reduced right ankle-brachial index (ABI) of 0.27. Pulse volume recordings (PVRs) revealed diffusely damped waveforms within the right lower extremity. The left lower extremity was normal. The symptoms of threatened limb combined with imaging findings were consistent with arterial ischemia from external iliac artery compression by the newly placed venous stent.

The patient returned to interventional radiology for arterial angiography. The left common femoral artery was accessed and a 7 French destination sheath was advanced proximal to the right external iliac artery. Angiogram demonstrated severe focal narrowing along the right external iliac artery adjacent to the external iliac vein stent (Fig. 3). Two overlapping 5 mm diameter balloon-expandable covered stents were deployed and dilated up to 5 mm. Final angiogram demonstrated complete resolution of stenosis with brisk flow to the lower extremity (Fig. 3).

Post-procedure, there was return of 2 + doppler signals throughout the right lower extremity. The previously noted acute motor and sensory deficits resolved. In addition to continuing therapeutic lovenox, the patient was started on 75 mg of clopidrogel daily to maximize arterial stent patency. The patient experienced significant improvement of lower extremity swelling and pain. Comfort care measures were ultimately pursued given progression of cancer.

There has been a growing interest in complex venous interventions, owing to device improvements and increasing evidence to support catheter-based therapies for appropriately selected patients (Vedantham et al. 2017; Kishore et al. 2022; Mahnken et al. 2014). Given the growth and expanded use of venous interventions, it is important that proceduralists develop an awareness of unique complications so that they are appropriately managed. This report highlights s case of arterial ischemia secondary to extrinsic compression from venous stents. This appears to be a rare but increasingly recognized complication, as there has been a cluster of case reports published within recent years (Filtes et al. 2021; Trinidad et al. 2022; Subramaniam et al. 2021; Vasudev et al. 2019; Taylor et al. 2020).

Early recognition of this entity is important, as long-term sequelae from untreated limb ischemia can be devastating. One report describes the need for transfemoral amputation and subsequent issues with wound healing which required additional surgery (Trinidad et al. 2022). Reported symptoms from this process are consistent with threatened limb, including pallor, pain, diminished pulses, and motorsensory loss (Filtes et al. 2021; Subramaniam et al. 2021; Taylor et al. 2020).

In almost all published cases where ischemia was detected early, endovascular stenting of the compressed artery was effective for revascularization. We thus recommend angiography and stenting as the primary initial therapy. Since delayed diagnosis can have serious morbidity, there should be a low threshold to bring the patient to the angiography suite. If the situation is not emergent and the clinical picture is unclear, CT angiogram can be considered to rule out other potential causes of ischemia, such as hematoma or distal thromboembolism. Vascular surgery should also be engaged early if there is suspicion for compartment syndrome or the need for open revascularization (Filtes et al. 2021; Subramaniam et al. 2021; Vasudev et al. 2019; Taylor et al. 2020).

Although the risk factors for this phenomenon have not been rigorously studied, there are common themes within the published literature. Case reports have described this complication among patients with active pelvic malignancy, previously irradiated fields, prior pelvic surgeries, or other inflammatory processes (Filtes et al. 2021; Trinidad et al. 2022; Subramaniam et al. 2021; Vasudev et al. 2019; Taylor et al. 2020). These factors likely result in decreased compliance of the perivascular soft tissues, ultimately transferring greater compressive forces on the artery by adjacent venous stents. Proceduralists should thus have a greater index of suspicion for this complication among patients with these clinical characteristics.

While there is no clear trend, use of certain stents may contribute to the occurrence of this complication. Case reports have described this complication in the presence of both covered and uncovered stents, as well as self-expanding and balloon-expandable stents (Filtes et al. 2021; Trinidad et al. 2022; Subramaniam et al. 2021; Vasudev et al. 2019; Taylor et al. 2020). The stent used in our procedure was a Viabahn VBX balloon-expandable covered stent graft (W. L. Gore & Associates Inc, Newark, DE, USA). This stent was utilized in lieu of typical self-expanding venous stents because in our experience at a large cancer center, self-expanding stents frequently lack the sufficient radial force needed to treat tumor compression (especially in previously irradiated fields). We found that when only venous stents were used in similar cases, completion venography and intravascular ultrasound would demonstrate severe compression of the venous stent, thus placing the patient at high risk of re-thrombosis and necessitating the use of balloon-expandable stents with greater radial force. In addition, covered stents may have an added theoretical benefit of limiting local vascular invasion and thrombosis from aggressive tumors.

This complication was also observed when using Viabahn self-expandable stents during a similar case at our institution, as well in another case report (Subramaniam et al. 2021). These stents are technically designed for arteries, so they are relatively less flexible compared to venous stents. This combined with the noncompliant surrounding soft tissues may have led to excessive forces on the adjacent artery. This hypothesis is confounded by the fact that newer generation dedicated venous stents, which are designed to balance flexibility with radial force (Shamimi-Noori and Clark 2018), have also been associated with this complication (Filtes et al. 2021). The heterogeneity of stents associated with this complication thus suggests that underlying patient factors, rather than stent selection, are more important predictive factors. Regardless, this highlights the need for operators to have a greater index of suspicion for arterial occlusion when using stents with greater radial force are required to achieve patency in the venous system. More data is required to optimize stent selection in this setting.

The most frequently reported location of arterial compression after iliocaval stenting is the external iliac artery (Filtes et al. 2021; Subramaniam et al. 2021; Vasudev et al. 2019). Compression of the external iliac artery, rather than the common iliac artery or aorta, is likely due to multiple factors. The external iliac vein runs immediately adjacent to the artery, therefore increasing the likelihood of compression along this segment. The external iliac artery is also tightly surrounded by bone, pelvic muscles, lymphatics, and fascia which limit the baseline compliance of surrounding soft tissues. The external iliac artery is also smaller in caliber than the more proximal vessels, so it may be less capable of withstanding compressive forces from adjacent strictures. Given the susceptibility of the external iliac artery to compression, we recommend placement of external iliac venous stents only when clearly necessary. If external iliac vein stenting is warranted, placement of oversized stents should be limited.

This case report raises the question of whether routine assessment of lower extremity arterial supply should be performed prior to any iliac vein intervention. In our case, checking pulses prior to our venous intervention was valuable for rapidly diagnosing an arterial problem, since a dramatic loss of arterial doppler signal should not occur after a venous intervention. Since this is easy to perform, we recommend routine perioperative pulse checks for patients who undergo complex venous interventions. Intra-procedural pulse-oximetry monitoring of the toes may also be useful to quickly assess for decreased arterial perfusion and guide whether immediate angiography should be performed (Kwasnicki et al. 2022). The use of intravascular ultrasound after stenting may also be beneficial in identifying arterial compression during the procedure. Further study would be useful to validate these tools.

Although severe stenosis may manifest with clinically overt signs of threatened limb, more subtle effects of ischemia may go undiagnosed or misattributed to other etiologies, such as post-thrombotic syndrome. Thus, more objective assessments of arterial perfusion using non-invasive vascular studies including ABIs, PVRs, and doppler ultrasound should be considered in high risk patients. Rigorous peri-procedural evaluation of patients with peripheral artery disease or diabetes may also be beneficial to identify subclinical cases of decreased arterial perfusion, so that future issues with wound healing can be minimized. Ultimately, more research is warranted to effectively diagnose and manage this complication.

## Conclusions

Extrinsic arterial compression secondary to venous stent placement is a rare but increasingly recognized complication following iliac vein stenting. Potential risk factors include patients with active pelvis malignancy, prior radiation, or scarring from surgery or other inflammatory processes. In cases of threatened limb prompt treatment with arterial stenting is recommended minimize serious long-term sequelae. Further study is warranted to optimize detection and management of this complication.

## Data Availability

Data sharing is not applicable to this article as no datasets were generated or analysed during the current study.

## Abbreviations

IVC: Inferior vena cava
ABI: Ankle-brachial index
PVR: Pulse volume recording
